# Patient Perspectives on Hospital Falls Prevention Education

**DOI:** 10.3389/fpubh.2021.592440

**Published:** 2021-03-16

**Authors:** Hazel Heng, Susan C. Slade, Dana Jazayeri, Cathy Jones, Anne-Marie Hill, Debra Kiegaldie, Ronald I. Shorr, Meg E. Morris

**Affiliations:** ^1^La Trobe Centre for Sport and Exercise Medicine Research, La Trobe University, Bundoora, VIC, Australia; ^2^Healthscope Ltd, Melbourne, VIC, Australia; ^3^Faculty of Health Sciences, School of Physiotherapy and Exercise Science, Curtin University, Perth, WA, Australia; ^4^Faculty of Health Science, Youth and Community Studies, Holmesglen Institute, Melbourne, VIC, Australia; ^5^Geriatric Research Education and Clinical Center (GRECC), Malcom Randall Veterans Affairs Medical Center, Gainesville, FL, United States; ^6^Department of Epidemiology, University of Florida, Gainesville, FL, United States

**Keywords:** focus group, patient education, qualitative study, falls prevention education, hospital falls

## Abstract

Hospital falls remain an intractable problem worldwide and patient education is one approach to falls mitigation. Although educating patients can help their understanding of risks and empower them with prevention strategies, patient experiences of hospital falls education are poorly understood. This study aimed to understand the perspectives and preferences of hospitalized patients about falls prevention education. Three focus groups were conducted in Australian hospitals. A phenomenological approach was used to explore patient perspectives and data were analyzed thematically. The focus groups revealed that most people did not realize their own risk of falling whilst an inpatient. Experiences of falls prevention education were inconsistent and sometimes linked to beliefs that falls were not relevant to them because they were being cared for in hospital. Other barriers to falls mitigation included poor patient knowledge about hospital falls risk and inconsistencies in the delivery of falls prevention education. A strong theme was that individualized, consistent education, and small interactive groups were helpful.

## Introduction

Empowering patients to prevent falls whilst in hospital is a clinical priority world-wide, yet surprisingly little is known about how best to educate people to mitigate hospital falls risk. Patient education is often part of a multifactorial approach to falls prevention (1–3). Patients are educated about different falls prevention strategies including using the call bell to seek help, wearing safe footwear, using assistive devices such as walking frames when prescribed, avoiding multi-tasking and waiting for assistance when mobilizing (1, 4). The published research shows that patient education can be effective in reducing falls-related outcomes, whether as part of a multifactorial program or as a single intervention (1–3, 5, 6). A recent scoping review recommended that patient education programs should be designed based on educational principles and health behavior change models (4).

In hospitals, older adults are at higher risk of falls due to a variety of factors (7, 8). These include changes in their mobility and balance associated with their medical condition or surgery, an unfamiliar environment, medications, co-morbidities, cognitive impairment or a history of falls (7, 9). Some patients take risks based on an overly-confident perception of their balance and mobility, or a belief that because they are being cared for in hospital they are protected from falls (10). This discrepancy between their perception and actual level of capability and function can lead to an increase in falls risk (10–12). Patient education is one strategy that aims to reduce this discrepancy, empowering people to reduce their risk and encourage adherence to falls prevention strategies (13). According to the Capability, Opportunity, and Motivation Model of Behavior (COM-B) model of health behavior change (14), education can improve psychological capability, as well as encourage reflective motivation. The COM-B model has been extensively used to guide behavior change interventions. It is based on the idea that for effective health behavior change, individual capability, opportunity, and motivation need to be present. This empowers them to adhere to falls prevention strategies and reduce their risk of falls.

While there have been studies on patient experiences of falls in hospital, as well as their experiences of falls prevention interventions, there is limited research into their views of falls prevention education (15–17). A qualitative study by Shuman et al. (17) explored patient perceptions of falls prevention interventions in general. They noted that many people initially denied receiving explicit falls prevention education, yet later described being given advice and instruction for preventing falls. This may have indicated a lack of engagement with falls prevention education. In other settings such as long-term care facilities, there is limited research into patient education as a single intervention (1). In the community, Smith et al. (18) demonstrated that patient education was effective for managing fear of falling. Further exploration of the views of patients about falls prevention education in hospital could provide valuable insight into which interventions to prioritize. Understanding patient perceptions, expectations and experiences of falls prevention education shall inform the design of future education programs.

The main aims of this qualitative study were to: (i) explore hospital patient experiences of falls prevention education; (ii) investigate barriers and facilitators to their understanding of, and adherence to, hospital falls prevention education programs; and (iii) understand the preferences of patients for falls prevention education.

## Methods

This study received ethics approval from the La Trobe University Human Research Ethics Committee (HEC19373). All participants provided written informed consent prior to participating in the focus groups. Each participant was assured of their anonymity and the confidentiality of the study. All recorded data in transcripts and demographic forms were de-identified by number. This study was registered in the Australian New Zealand Clinical Trials Registry (trial number: ACTRN12620000033943). The study was reported according to the Standards for Reporting Qualitative Research (SRQR) (19).

Phenomenology was chosen as the theoretical framework (20–22). This qualitative approach draws from the lived experiences of participants to develop understanding based on their experiences and perceptions. The approach can utilize thematic analysis of participant perspectives to identify themes linked to their data (21–23).

The following steps were followed to promote rigor of study methods and to minimize risk of bias: (i) use of an a priori methodology; (ii) involvement of at least two independent researchers for data analysis; (iii) recruitment of hospital participants with experiences relating to the research questions; (iv) audio-recording and accurate transcription of interview data; and (v) ensuring that emergent themes were supported by relevant quotations from participant data (24, 25).

The focus group design selected for this study was advantageous for several reasons. It allowed for interactions between group participants to elicit new insights (26, 27). These groups were facilitated by an experienced qualitative researcher (SCS) who was not known to any of the participants. A note-taker (HH) was present during these focus group sessions to capture non-verbal or emotional responses, as well as to document observations.

The study team included members who were experts in hospital falls prevention (MEM, PhD; A-MH, PhD; RS, PhD) and patient education (DK, PhD). In addition, SCS (PhD), MEM, and DK were experienced in qualitative research methods. MEM had extensive research project management experience and supervision expertise. DJ (PhD) and RS (PhD) assisted with data analytics, and HH (M Physio) and CJ (MBA) provided clinical expertise.

The eligibility criteria for participation in the focus groups were: (i) hospitalized adults (>18 years old) who could speak, read, and understand English; and (ii) inpatients in Healthscope hospitals, Australia. Patients were excluded if they were: (i) scheduled for discharge prior to commencement of the focus group; (ii) medically unstable; and (iii) did not stay overnight in hospital (e.g., emergency department presentations, day surgery patients or day oncology patients). Patients were also excluded if they were judged to have limited cognitive capacity to participate in a focus group. This was assessed by nurse unit managers using their clinical judgement.

Recruitment of participants was conducted via purposive sampling (28, 29) from two hospital sites belonging to the same private health network. Patients were recruited from five wards including acute medical, acute surgical, orthopedic, and geriatric rehabilitation. In the Australian private hospitals where patients were recruited from, there was a nationwide policy for falls prevention, which the nursing team and other health professionals followed. This policy provides details on falls prevention governance and systems, methods for capturing and auditing falls data, evidence-based clinician education, patient education, environmental adaptations, exercises and physical activities, diet, medication monitoring, and tailoring treatment plans to individual needs. The policy emphasizes the importance of encouraging patients to use the call bell when needing assistance. The use of safe footwear, glasses and assistive devices are highlighted, as well as how to adjust falls mitigation for cognitive impairment or multi-morbidity. A researcher (HH) liaised with nurse unit managers to assist with recruitment. The nurse unit managers would identify potential participants according to the eligibility criteria and screen for patients with impaired cognition based on their clinical judgement. The same researcher (HH) approached the potential participants to explain the purpose of the study and to provide them with a participant information statement. After 24–48 h, HH returned to discuss participant interest and to obtain informed consent.

Patients who agreed to participate signed a consent form and completed a demographic questionnaire. This included questions about falls history in and out of hospital, current mobility, and past medical history. Recruitment ceased when researchers determined that data saturation had been reached through careful reviewing of all transcripts. Saturation occurred when no new themes or ideas emerged following analysis of transcripts (30, 31). Eleven patients were included in the final sample.

Data collection occurred in Australian private hospitals. The focus groups were conducted at the hospitals in which the participants were inpatients. The facilitator (SCS) guided the discussion with a list of interview questions that were derived from a scoping review (4) and from expert opinions from members of the study team (Table 1). All sessions were audio-recorded and transcribed verbatim by an independent transcription service. Participants were designated a number which replaced names in the transcripts and ensured anonymity. Demographic questionnaires, which were completed prior to participating in the focus groups, did not contain any identifying details. There was a debriefing session between the facilitator and note-taker immediately after each group and a summary was circulated to the research team. Physical and digital data were stored securely in a password protected secure University research drive and locked filing cabinet in a secure University office.

**Table 1:** Interview guide.

1. Do you believe you are at risk of falling in hospital? • If yes, why; if not, why not? • Do you feel your risk of falling is any different in hospital compared to at home? 2. Have you had any information about preventing falls while you are in hospital? • If yes, what was it like? Who provided it? Where and when was it provided? • Was it helpful? If yes, why; if not, why not • What did you think about the information given to prevent falls?
3. Have you experienced a fall during any stay in hospital? 4. Did you receive any advice or information about preventing falls after your experience • Do you attend exercises in hospital? What are the exercises? (Balance vs. Strength) 5. Have you used that advice? • If yes, why; if not, why not?
6. Did you understand the advice or information given about preventing falls? • If yes, why; if not, why not? 7. If you were going to be taught about falls prevention in hospital, what information would be helpful? 8. What was the best advice you received while in hospital about falls prevention?

## Data Analysis

Thematic analysis was used to identify and develop themes (20). This analytic framework allowed for in-depth exploration of the data to identify and analyze response patterns (20, 32). An inductive approach was taken (20) as there was limited evidence around the topic of patient perspectives about hospital falls prevention education. One researcher (SCS) ensured accurate transcription of the data by comparing the audio recordings and verbatim transcripts. Two researchers (SCS, HH) independently examined and read the de-identified transcripts multiple times to ensure familiarization and to identify codes and categories. Coded data were presented in spreadsheets and documents and were discussed in iterative stages. Categories and emerging themes were discussed between researchers via documented face-to-face or videoconference meetings. A third researcher (MEM) was consulted if there were any discrepancies in opinion until consensus was reached. Once final themes and sub-themes were identified and consolidated, relevant participant quotations were selected to help to determine each theme.

## Results

### Characteristics of Participants

Between December 2019 and February 2020, a total of 11 of 33 potential participants were interviewed in three focus groups in private hospitals. Twenty participants were excluded as they declined to participate, while two were excluded due to unexpected early discharges. There were three participants from an acute medical ward that formed focus group 1. There were another three from an orthopedic rehabilitation ward that formed focus group 2. Five patients from three rehabilitation wards formed focus group 3. The mean age of participants was 78.4 years (SD 15.32, range 37–92). The mean duration was 51.21 min (SD 8.37). Three participants were male and eight were female. Six participants reported at least one fall in the past 12 months. Five out of six were over the age of 70 years and four out of six had multiple co-morbidities and medications. Four participants, one of which did not have multiple co-morbidities, experienced more than one fall in the past 12 months. Of the five fallers, two had fallen whilst in hospital (age 37 and 92 years old). Patient demographics can be found in Table 2.

**Table 2:** Patient demographics.

**Participant characteristics**	**Participants (*n* = 11)**
Mean age, y (SD)	78.4 (15.32)
**Gender**	
Female	8
Male	3
**Number of fallers**	
In past 12 months	5
Who fell in hospital	2
**Number of co-morbidities**	
None	1
1–4	4
5–8	1
9–12	4
>12	1
**Number of medications**	
None	0
1–4	5
5–8	4
9–12	1
>12	1
**Reason for hospitalization**	
Medical diagnoses^*^	2
Orthopedic	4
Respiratory	1
Other surgeries^**^	4
**Mobility status**	
Without gait aids	4
With gait aids	7

* *includes digestive system disorders, infections, and renal disorders*;

** *other than orthopedic surgeries*.

The overarching theme to emerge was that most hospitalized adults did not realize their heightened falls risk whilst in hospital. Four main themes were identified: (i) there was a mismatch between perceived risk and actual risk of hospital falls; (ii) there was inconsistency in patient education experiences whilst in hospital; (iii) there was value in educating and empowering patients to prevent their own falls whilst in hospital; and (iv) there were individual preferences for education delivery.

### Theme 1: Mismatch Between Perceived Risk and Actual Risk of Hospital Falls

All of the participants in our focus groups reported feeling safer in hospital than at home. They typically attributed this to the hospital environment being modified for safety. They also felt cared for and perceived that staff were available to supervise and assist them when they were unsteady or unable to mobilize independently (Box 1). One participant felt safer because they did not need to complete their usual tasks of daily living, such as cooking, cleaning and working. Most participants did not know exactly what they should do to prevent falling whilst in hospital. Over half had limited knowledge about the different contributing factors to falls in hospital. Many had only a basic understanding of factors that increased the risk of falls whilst hospitalized (Box 1).

Box 1 **Mismatch between perceived risk and actual falls risk**

“*I have had one [fall]… I wasn't supposed to walk without help… my sons would tell you because I'm bloody-minded” FG1, P1, lines 255, 259, 262*.

“*Not really [at risk of falling], no. I mean, bit generally. I guess, as I've come out of each surgery and been weaker, that's probably where the risk's at. You know, when my blood pressure was low and I thought I could walk a bit more than I could” FG1, P3, lines 57–58*.

**“***I've had the fall. Last time I was in hospital, I was feeling very sort of faint and I got up on my own to go to the toilet and I was attached to all the poles and stuff—and I was allowed to go up on my own, like the nurse had said I was allowed to—and I just sort of fell” FG1, P3, lines 214–217*.

“*I think you're safe [in hospital] because there's always people around you, if you go for a walk, and ask you if you are alright” FG2, P4, lines 27–29*.

“*I have a chair here and I find it fantastic because there's a very good rail and the chair that is light to move and with the walker, I have no trouble at all. And I don't find the floor slippery because it's got a texture” FG3, P10, lines 150–153*.

### Theme 2: Patient Education Experiences Were Inconsistent

Falls prevention education varied between participants. Different methods of falls education delivery were also reported. Less than half of the participants described receiving verbal advice or instructions from staff (usually nurses) such as using the call bell, or not rushing to mobilize. Four reported only receiving fall prevention brochures with no face-to-face discussion on how to use them or what to focus on. Posters on falls prevention provided advice that was followed by some participants. Some people received physiotherapy and occupational therapy falls assessments, yet for others this did not occur. Four of the 11 participants had attended a falls education group whilst in hospital. The falls education groups were most often reported to be conducted by an occupational therapist and often focused on falls prevention after hospital discharge. Two participants had home visits by the occupational therapist to identify hazards and recommend environmental modifications for their home post-discharge. There were also inconsistencies in the timing of delivery of falls prevention education. Some patients were given falls education brochures on admission or prior to discharge yet had no explanation from clinicians regarding the content or how to apply the information. Many participants recognized the inconsistency and preferred the provision of standardized falls education (Box 2).

Box 2 **Patient education experiences were inconsistent**

“*haven't been given them [falls brochure] but they are along there [hospital corridor] and I've read them… I was looking at all the pamphlets and I took that one and I read the information” FG1, P1, lines 179, 181*.

“*They just tell me ‘think about it and take your time, no rush'" FG1, P2, line 523*.

“*I saw the pamphlet, but I didn't look any further than the front cover. I didn't take it and read it” FG1, P3, lines 207–208*.

“*well, we had a session on that very screen about a week ago, showed a film on how to get up, but it's only a help, it doesn't guarantee you anything” FG2, P6, lines 317–319*.

“*Well it's reasonably comprehensive [the falls brochure]. I think it's—and it was drawn to my attention the first day that I came here … it was put down and said, ‘We strongly urge you to read this.'" FG3, P8, lines 605–615*.

“*It seems that there is somewhat inconsistency of those things like that because whereas with this particular one, I was given it on the very first day, another brochure which is particularly important in my case was how to avoid doing things that might dislocate your hip wasn't given to me until a week after I'd been here” FG3, P8, lines 630–634*.

“*I was lucky in that the person who showed me into the room went to the trouble of drawing these brochures to my attention and suggested I really ought to read them. But it seems from what I've heard that it's not a general policy” FG3, P8, lines 637–640*.

### Theme 3: Empowering Patients to Prevent Their Own Falls Whilst in Hospital Was Valued

Empowered patients within the focus groups appeared to be more receptive and willing to adhere to falls prevention education whilst in hospital. A history of strength and fitness seemed to predispose many participants to understand the importance of exercise in reducing falls (**Box 3**). Prior experience of an active or healthy lifestyle was perceived to promote recovery and rehabilitation. Staff modeling of safe behavior also allowed participants to identify hazards and increased their mindfulness of falls risks. Other factors that promoted empowerment included insight into their physical status associated with their medical condition, knowledge about consequences of a fall, reminders such as posters and pamphlets, and participation in group falls education (Box 3).

Box 3 **Empowering patients to prevent their own falls whilst in hospital**

“*the first couple of days (in ICU), when I was walking, yes, I was a bit more scared of falling over because I was very, very weak—but I did have a nurse holding me” FG1, P3, lines 65–67*.

“*having just had a hip replacement, I'm very, very conscious of the dangers of falling so I'm always making sure I'm conscious where I'm putting my feet” FG3, P8, lines 54–57*.

“*one thing that … did influence my behavior in this hospital was the signs up saying that shoes are safer than socks. So as a result of that, when I get up in the night to go to the toilet … I put these slip on shoes on rather than walk across in my socks. And that was a direct result of seeing that educational material posted up here” FG3, P8, lines 210–216*.

“*we're involved in an old people social club, you hear stories of people who have fallen and the disastrous consequences that can happen. And that has led me to be very, very conscious of it” FG3, P8, lines 185–187*.

“*and she's walked for probably 30 years, she's 90. And went into hospital and it healed itself, because it was a fracture, and she was in traction I think for a while. And then she had rehab. She's back walking with us because she had all that fitness before. She's just brilliant” FG3, P7, lines 805–809*.

“*I'm told I've made an incredibly recover[y]. But I too have always done exercises every day and I've walked for 45 min every day and sometimes much longer. It's just been always part of my life” FG3, P10, lines 367–372*.

“*every time one of them [staff] sees, as you know when you're drinking water you spill a drop and it goes here, every single member of staff who has come in and spotted it has cleared it and said, ‘That's dangerous.' And they take a cloth and wipe it and put it away. So that in the way has impressed on me again.” FG3, P10, lines 291–299*.

There were several perceived barriers to patients understanding their risk and preventing their own falls whilst in hospital. Knowledge deficits about falls prevention were a major barrier that could potentially be addressed with education. Four participants were reluctant to interrupt or burden staff by calling for assistance when they needed to mobilize. One person felt that supervision or assistance was unnecessary and had a misperception that they were independent despite significant disability. For some, reduced motivation, and poor understanding of the importance of exercise were barriers to engagement in strength and balance therapies to help reduce falls. Over a third of participants reported poor retention of previous falls prevention education or a gap in their knowledge about risk factors. One younger adult explained that falls education was not relevant to her because she thought she was not at risk of falling. Some older participants perceived falls to be inevitable with aging. Therefore, they did not always prioritize falls prevention practices (Box 4).

Box 4 **Poor patient knowledge was a major barrier to falls prevention**

“*I believe that aging, in itself, as you get older, quite a number of people start losing their balance, apart from other causes, just getting old is a part of it” FG1, P1, lines 169–171*.

“*perhaps I didn't do them [exercises] long enough, I don't know, but I haven't felt that they have improved my balance at all” FG1, P1, lines 513–515*.

“*I do usually wear the slippers if I'm walking any further than my room. But if it's in my room, I'm just bare feet” FG1, P3, lines 78–80*.

“*I think, ‘Oh, someone's probably sicker than me so I can probably do it myself'" FG1, P3, lines 594–596*.

“*I don't see falling as something that I need to be scared of. But I imagine, as I get older, that is going to something that is going to be an effect. So when I'm in hospital though, it's hard to change that mindset” FG1, F3, lines 356–360*.

“*I don't want to cause strife for the girls to come along unnecessarily, if you don't think it's necessary” FG2, P5, lines 660–661*.

“*I've got to admit, I've got lazy at home…I stopped doing the exercises gradually, and I paid the penalty for it… I should have kept on going them. I won't do that again” FG2, P6, lines 187, 189, 192*.

“*if I can see they're all busy I'll try and work it that I wait until I feel that it's more convenient, if it's not urgent … I don't sort of just press the bell if I can see them scurrying around doing other things” FG3, P9, lines 483–487*.

### Theme 4: Individual Preferences for Educational Delivery Mode Varied

The participants reported individual preferences for the content and way in which education was delivered. Most people expressed a desire for consistent messages from all staff and for education to also be tailored to individual needs. For the content of falls prevention education, many participants preferred to know more about the consequences of falling, what to expect and do post-operatively, strategies to manage falls and options for information while hospitalized. Some wanted more direction on how to consult physiotherapists, nurses, and occupational therapists for information. Six participants advised that having falls prevention reminders such as posters and resources would be helpful. Small group interactive education was recommended by some people as a useful way of receiving falls prevention education (Box 5).

Box 5 **Participant preferences for falls prevention education**

“*that would be of help, yes [information about improving leg strength]. That's one of the reasons I go to my strength training class” FG1, P1, lines 412–415*.

“*[Brochure] probably would help it, yeah. And my mind having more flow and I read it and I remember more” FG2, P2, line 527–530*.

“*it's about, post-operatively, what to expect … I don't actually think about the correlation between the fact that I'm under all these drugs to the fact that that's going to make me less balanced and steady on my feet. Like I don't actually see that correlation naturally” FG1, P3, lines 546–550*.

“*I think the trouble is, in any sort of organization, the people who have the information and know what should be done, it's so obvious to them and they understand it so well, that they don't necessarily realize that the people who need it don't know it all and have never heard of such a thing” FG3, P8, lines 672–676*.

“*I am an older person and even though it may not relate to me at the moment, I think I'd like all that information because it makes me aware. I'm just as likely to fall as perhaps not somebody that's had a physical problem” FG3, P7, lines 780–784*.

“*thinking about the sort of warning sign which I think would be useful to have, just a simple thing like a fall could kill you, watch your step, because I think that as we get older, we tend to forget just how serious a fall could be … So the seriousness of it perhaps could be emphasized a bit more” FG3, P8, lines 767–774*.

“*Well I found out more today than I have in the last 2 weeks that I've been here” FG3, P11, lines 898–903*.

## Discussion

Many of the hospital patients that we interviewed were not aware of their risk of falling whilst being an in-patient, despite having advanced age, underlying medical conditions, multiple medications, previous falls or recent surgery. The National Institute of Health and Care Excellence guidelines advise that hospitalized adults aged 65 years or older and hospitalized adults aged 50–64 years with an underlying condition have a heightened falls risk (8). Falls risk is also increased when people have multi-morbidity, cognitive impairment, polypharmacy or a history of recent falls (7, 8). This is reflected in our study where participants who reported multiple falls had multiple co-morbidities and medications. There was a mismatch between this evidence and what many hospitalized adults believed. In agreement with global research, the participants in our trial did not always identify falls as a serious or relevant problem for themselves (33, 34). Some thought that falls only occurred in very old or very disabled people, which is contrary to the published literature (1, 8). Another study by Anderson, Postler (35) showed that patients under the age of 49 had the highest percentage of fallers across 12 months in a tertiary hospital.

Some older adults, including those in our study, perceive falls risk as primarily due to external or environmental hazards such as spills, rugs, cords or clutter (34), rather than personal factors. Whilst in hospital, some people also have a false sense of security and believe that the environmental design of a hospital and the presence of staff protects them from falling (16). They may not be aware of the evidence showing that falls occur in hospitals even with the availability of staff, call bells and modified environments (7, 36). Some patients also take risks due to a limited understanding of falls risk factors (10). Others assume they will maintain their pre-morbid level of function and risk without considering the reasons for their hospitalization (16, 37). Together, these misunderstandings heighten hospital falls risk as well as reduce patient awareness of the need for falls prevention education.

The methods used for hospital falls education varied considerably for the participants in our focus groups. Consistent with the scoping review by Heng et al. (4) the interventions included face-to-face advice from nurses and other health professionals, posters, pamphlets, brochures, bookmarks, occupational therapist led falls prevention groups, and educational videos for patients. The participants in our trial also reported variability of timing, delivery and follow up of patient information and education, which aligned with the findings of Lee et al. (38). Inconsistency in falls prevention education could stem from several factors. The resources required for falls prevention education are modest, yet their cost may have restricted access and availability, as shown by two studies (39, 40). Time constraints or a lack of experience in providing falls prevention education have been shown by other investigators to be associated with unwanted variability in what is delivered (41). Patients in rehabilitation settings may receive different patient education compared to acute settings.

Skelton (42) suggested that staff sometimes follow a generalized falls education protocol, with lack of individualization of falls mitigation interventions. Theoretically, a lack of patient-centeredness in falls prevention education could reduce empowerment and adherence to prevention strategies (43). Moreover, inconsistencies in the provision, content and follow-up of falls prevention education could lead to mixed messages (38). Patients often need to hear the same consistent message from more than one staff member in order to take heed of the strategies. Receiving differing advice, or a lack of advice, from staff members, could lead to uncertainty about how to prevent a slip, trip or fall. When patients are not aware of the gaps in their knowledge, it is difficult to achieve changes in health behavior (14). In addition, some of the focus group participants may not have recalled the education provided. Falls prevention education specific to a hospital environment is likely to require repetition as retention of strategies may diminish once a patient is discharged (12, 44, 45). Prior knowledge may also be incorrectly assumed by nursing staff for patients who have had a previous hospital stay.

Empowerment of patients to prevent their own falls whilst in hospital was a strong theme in our focus groups, with positive empowerment identified as a facilitator in patient adherence to falls prevention education. Patients did not always realize that falls were likely, why falls occurred or how to prevent them, and this was a major barrier. Making this clear appears to be an important step in empowering them to stay safe (10, 11, 46, 47). In addition, some individuals were better able to learn and apply new knowledge than others. In this respect, past behavior, intrinsic motivation and intentions (48) are known to predict future falls prevention behaviors (49, 50). The participants who reported past experiences with exercise and fitness appeared to have greater understanding of falls risk factors and stronger intentions to implement advice from falls education programs. We also found that empowerment was linked with intentions. Jacobs et al. (51) reported that changes in intention are closely associated with changes in physical behavior. Some participants thought that falls were inevitable with age and did not prioritize falls prevention (34). For some there was a low self-perceived risk of falling, which has been associated with low engagement in falls prevention activities (12).

Another facilitator was staff modeling of safe behaviors to prevent falls. Staff modeling safe behaviors was reported by several participants to assist patients to prevent falling. This included modeling how to keep the bathroom floor dry. It also included modeling how to walk safely using an assistive device. Modeling has been shown to increase motivation as advocated in Michie's et al. (14) health behavior change model. Assisting staff to be responsive to patient requests appears to be related to implementing a fall-free environment. Hospital patients are conscious of the busy nature of hospitals and are often reluctant to ask for help when they perceive that staff are occupied. Clinicians can be encouraged to be aware of their body language, voice tone and language when delivering falls education and reassuring patients of their availability to assist (37).

Patient preferences for falls prevention education included individualized interventions and consistent standardized information from all staff. Simplified and consistent messaging has been utilized effectively in health promotion and communication worldwide (52). Small group discussions have been shown to be an effective way to share and build knowledge (53, 54). In agreement with Peel and Warburton (55), our focus groups also highlighted that interactions between participants can generate new insights, allowing for deeper understanding of falls and falls risk in hospital. Small group interaction allows for peer education, which can provide a sense of connection, social benefits whilst being cost-effective (56). This can facilitate engagement and improved uptake in knowledge (56). These preferences reflect aspects of the behavior change model (14). Future clinical practice and hospital falls prevention education programs would benefit from taking these patient perspectives into account. These programs will be able to empower patients further by aligning content and delivery with patient views.

There were several limitations of this qualitative study. All participants were in private hospitals and the results might not generalize to the public (government funded) hospital system. The study was limited to English speaking participants. In addition, data were collected in Australia and the findings might be different for hospitalized patients in other countries. The study was on adults in hospital and are not applicable to pediatric patients or outpatients. For outpatients, some of the findings relating to a false sense of security in hospital could be different. Although patients without cognitive capacity were excluded from the study, these findings were based on unverified recollections that may be affected due to altered mental status in hospital settings. Especially in rehabilitation units, the goal to achieve independence in ambulation may be at odds with an overt emphasis on not falling (57). A limitation of this small study was that only five of 11 patients reported falling over in the last 12 months. In the development of a falls prevention education intervention, past history of falls is a key consideration.

## Conclusion

Hospital patients in this study had optimistic views about their risk of falling whilst in hospital, which did not always align with their actual risk. The under-estimation of risk was sometimes related to limited knowledge and variable experiences with falls prevention education. Factors facilitating understanding and adherence to falls prevention education included staff modeling of safe behaviors, falls prevention reminders, and tailored simple falls prevention advice to meet individual needs. Falls prevention education groups delivered whilst in hospital increased knowledge and capability. There were varied preferences for falls education delivery and a patient-centered multi-factorial approach appeared to be most successful.

## Data Availability Statement

The de-identified raw data supporting the conclusions of this article will be made available by the authors, without undue reservation.

## Ethics Statement

The studies involving human participants were reviewed and approved by La Trobe University Human Research Ethics Committee. The patients/participants provided their written informed consent to participate in this study.

## Author Contributions

MEM, HH, DK, and DJ conceived of this study. HH, SCS, DJ, DK, and MEM developed the design of this study. HH, SCS, DJ, and MEM carried out the focus groups. HH, SCS, and MEM analyzed the data and discussed the results with all authors. HH took the lead in writing the manuscript while all authors provided critical feedback. The final manuscript was approved by all authors prior to submission.

## Conflict of Interest

The authors declare that the research was conducted in the absence of any commercial or financial relationships that could be construed as a potential conflict of interest.
